# Deep-Fuzz: A synergistic integration of deep learning and fuzzy water flows for fine-grained nuclei segmentation in digital pathology

**DOI:** 10.1371/journal.pone.0286862

**Published:** 2023-06-23

**Authors:** Nirmal Das, Satadal Saha, Mita Nasipuri, Subhadip Basu, Tapabrata Chakraborti

**Affiliations:** 1 Deapartemnt of Computer Science and Engineering (AIML), Institute of Engineering and Management, Kolkata, West Bengal, India; 2 Deapartment of Computer Science and Engineering, Jadavpur University, Kolkata, West Bengal, India; 3 Department of Electrical and Computer Engineering, MCKV Institute of Engineering, Howrah, West Bengal, India; 4 University College London and The Alan Turing Institute, London, United Kingdom; 5 Linacre College, University of Oxford, Oxford, United Kingdom; TU Wien: Technische Universitat Wien, AUSTRIA

## Abstract

Robust semantic segmentation of tumour micro-environment is one of the major open challenges in machine learning enabled computational pathology. Though deep learning based systems have made significant progress, their task agnostic data driven approach often lacks the contextual grounding necessary in biomedical applications. We present a novel fuzzy water flow scheme that takes the coarse segmentation output of a base deep learning framework to then provide a more fine-grained and instance level robust segmentation output. Our two stage synergistic segmentation method, Deep-Fuzz, works especially well for overlapping objects, and achieves state-of-the-art performance in four public cell nuclei segmentation datasets. We also show through visual examples how our final output is better aligned with pathological insights, and thus more clinically interpretable.

## Introduction

Robust semantic segmentation of tumour micro-environment is one of the major open challenges in machine learning enabled computational pathology [1]. It is an important, but hard problem to solve: it is important, since it provides a spatial handle to the user to then further inspect the interactions of the segmented regions in their proper biomedical context; and it is hard, due to the high heterogeneity of cancerous histopathology images. Thus a key starting point to attack this problem in many such scenarios, is to localise the different cell nuclei types in the tumour micro-environment [2]. Given the generally circular/spheroid appearance of any type of individual cells, it is common to first localise these in a robust manner, and then try to group them into meaningful clusters. Past approaches on cell nuclei segmentation include (but not limited to): mathematical morphology [3–6], pixel classification [7], level sets [8], graph based segmentation method [9] and others. All these traditional segmentation approaches rely solely on hand-crafted features and these hand-crafted features may not be optimal, sometimes difficult to devise.

Though modern deep learning systems are powerful tool to achieve automatic feature representations, these systems often operate as context unaware black boxes, since these are entirely data driven [10]. For natural scene images, with enough training data for generalisation, one can achieve robust deep vision systems [11] that can be deployed for real life applications. However, for human patient facing biomedical imaging applications, mere high accuracy is not enough for such systems to be adopted in real life clinical settings, design choices need to be made upfront to make the decision process more transparent and trustworthy, and also the system design needs to be in alignment with biomedical insights for that particular task.

One way of achieving an interpretable model, is to design a pipeline that has a purely data driven deep learning module that produces a coarse output, and then a transparent fine-tuning module that is tailor-made for the particular task, that improves on the initial deep learning output. Such synergistic systems, if carefully integrated, can exploit the power of deep vision systems, and at the same time harness the open nature of more traditional computer vision and machine learning approaches. The objective of the present work is to achieve robust instance level cell nuclei segmentation, mainly targeting overlapping nuclei, from microscopic images. We used four public cell nuclei image datasets to validate the proposed method. Our proposed segmentation pipeline, Deep-Fuzz, falls in this new genre of systems, where we first have a U-Net based lightweight deep network that produces a coarse segmentation of cell nuclei in a purely data driven task agnostic manner. But then this output is fed into a fuzzy water flow based segmentation module, that is especially designed to work for overlapping cell nuclei boundaries, which is often the case for this type of images, and the generic deep network is not proficient at handling such cases [12, 13]. We demonstrate that this synergism helps to achieve better instance level segmentation results, even for the overlapping nuclei, and makes the total cell count for the image more accurate. The contribution of this paper is, thus, threefold.

1. We present a novel fuzzy water flow scheme that can take a coarse segmentation result of cell nuclei in digital pathology images, and then fine-tune them to generate highly robust fine-grained instance level segmentation output, even for overlapping cells, in a completely unsupervised manner, without requiring any further learning.
2. In the proposed segmentation method, Deep-Fuzz, we integrate the fuzzy water flow module onto a base deep learning framework configured with vanilla U-Net and Atrous Spatial Pyramid Pooling (ASPP). The deep learning model generates the coarse segmentation, which is then improved upon by the fuzzy water flow module. Deep-Fuzz produces state-of-the-art results on four public cell nuclei image datasets.
3. An additional benefit of Deep-Fuzz comes from the synergy between supervised deep learning and the unsupervised fuzzy water flow algorithm. The deep network is purely data driven whereas the fuzzy water flow algorithm is transparent and provides user access for handcrafted design, thus making the entire process more interpretable.

## Methods

The proposed segmentation pipeline Deep-Fuzz comprises of two phases 1) Coarse segmentation with U-net based deep learning network 2) Fine-grained segmentation of overlapping nuclei with fuzzy water flow method. In the following sub-sections we discuss the two phases in detail.

### Coarse segmentation with U-net based deep learning network

Deep networks have become popular in recent years since they work in a purely data driven, task independent manner and produce high performance when presented with sufficient amount of labeled data. However these systems are bulky and often opaque, and hence their accountability for tasks with high social consequences, like biomedical applications and computational oncology, is difficult to ensure. On the other hand, traditional image processing methods and some unsupervised machine learning methods can be quite lightweight and fast, with less data requirement as well. Since, these are hand-crafted by the designer, they are more amenable to fine-tuning and hence interpretation of the decision making process is easy, compared to a complex deep network with thousands of parameters. Hence in this work, we exploit the automated feature learning capability of the deep network based on underlying data distribution to get an initial coarse estimate, and then improve it for a more tailored segmentation using a bespoke novel algorithm based on fundamental underpinnings of the computer vision discipline. Due to the immense success of U-Net [14] in biomedical image segmentation, we selected U-net as our base framework. U-Net was especially designed to work with limited number of training samples which is true in case of biomedical images. Also we have used ASPP module [15] in the bottleneck of U-Net to increase the feature learning capability of the vanilla U-net by extracting multi-scale information with varying receptive fields. In the following, we describe the working of U-Net and ASPP module in brief.

**Fig 1:**
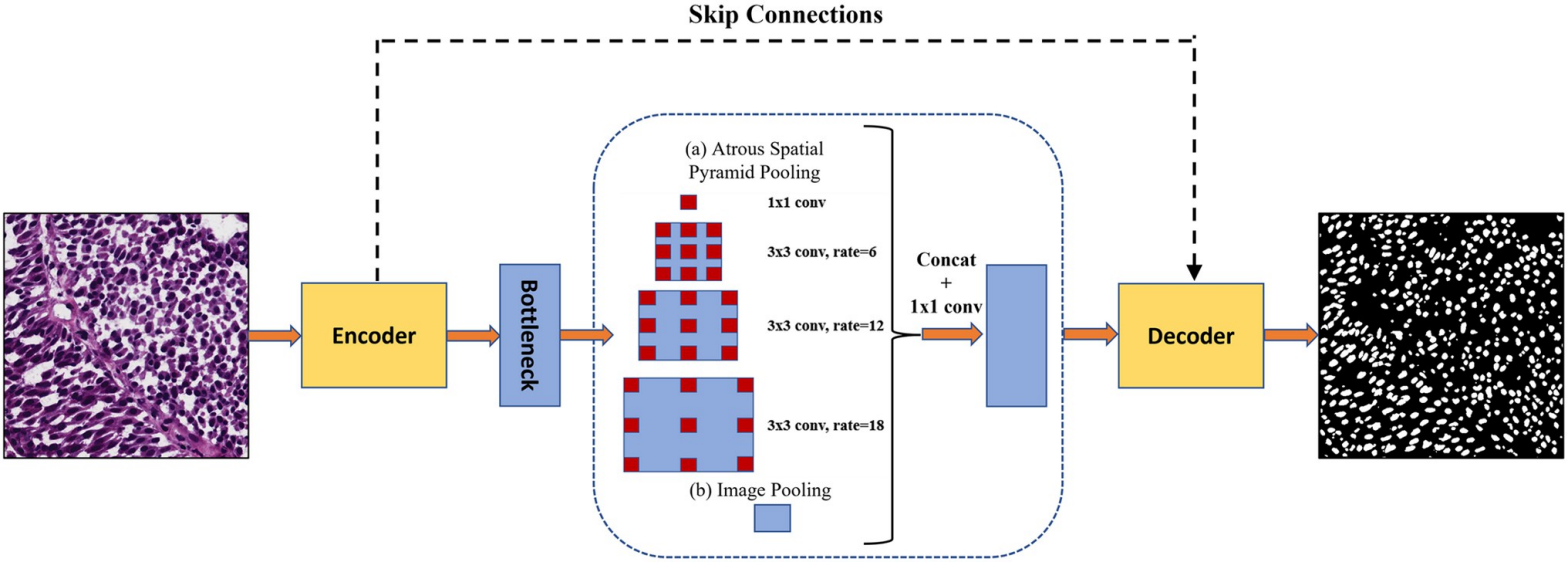
The working of U-net with atrous spatial pyramid pooling (ASPP) on a sample image. The ASPP module is inserted after the bottleneck of the Encoder-Decoder network. The feature map generated by the encoder is processed by using ASPP, and then, the result is fed into the decoder network for final prediction output.

U-Net consist of two part: encoder and decoder, connected by a bottleneck layer. The encoder part is the contracting path which follows usual steps convolutional network. Two 3x3 convolutions followed by a ReLU (Rectifier Linear Unit) activation function [16] and 2x2 max pooling operation with stride 2 are applied on each input image repeatedly for downsampling. The channels of the feature maps are doubled after each convolutional block. At each block in the decoder part the image is upsampled by using a 2 x 2 deconvolution and the corresponding feature map from the encoder part is concatenated using skip connection. This is followed by two 3 x 3 convolutions and passed through ReLU activation function. The number of feature channel are halved after each upsampling. In the final layer, a 1x1 convolution and Sigmoid activation function is applied to generate the output binary prediction image. The high-level properties of the input image are described in the bottleneck layer, which connects the encoder and decoder. The ASPP block in the bottleneck captures multiscale information by adjusting the size of its receptive field. ASPP consists of four parallel convolutional layers running at various atrous rates. Specifically, the ASPP module consists of (a) one 1x1 convolution and three parallel 3x3 convolutions with rates of 6, 12, and 18, respectively and (b) an image-level feature that is produced by global average pooling. The resulting features from all of the branches are bilinearly upsampled to the input size and then concatenated and passed through another 1x1 convolution. Fig 1 graphically represents the working U-net with ASPP block.

However, standard CNNs without any kind of post-processing are often unable to handle the case of segmentation of overlapping objects because the loss function minimized by the CNN is generally defined at the pixel level. This is obvious that the contribution of a single wrongly classified pixel to the global loss is minimal, yet it systematically leads to the combined segmentation of the objects.

### Fine-grained segmentation of overlapped nuclei with fuzzy water flow method

**Fig 2:**
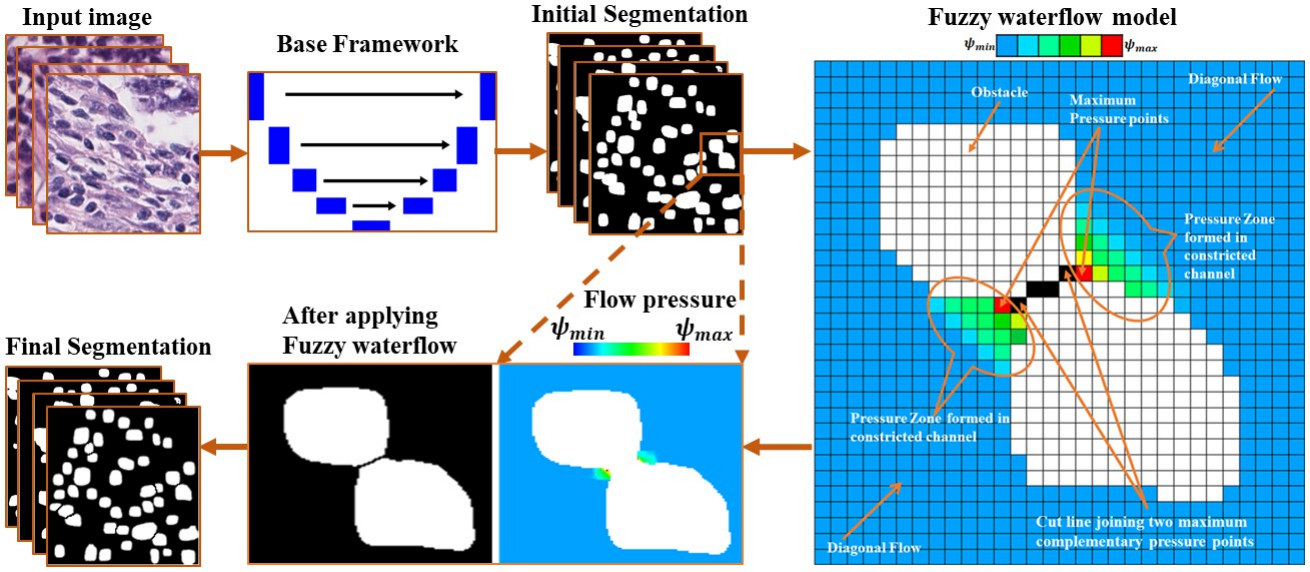
Workflow of the proposed segmentation pipeline Deep-Fuzz. Input images are passed to the base deep learning framework (U-net with ASPP block in the encoder-decoder bottleneck) for initial coarse segmentation. The proposed fuzzy water flow algorithm is applied to the coarsely segmented images for further segmentation of overlapping nuclei. The pixelated image on the right, a downsampled version of the selected overlapped nuclei image, illustrates how “flow pressure” increases when it passes through the constricted channels created by overlapping nuclei. The joining line of maximum flow pressure points in the two opposite directions creates the separation boundary. After the separation of overlapping nuclei, the final instance segmentation output is achieved.

The main methodological innovation of this work lies in the formulation of the novel fuzzy water flow scheme. It helps to fine-tune the output of the base deep network in a robust manner, especially for overlapping cell nuclei. Although water flow based segmentation was previously used for text line extraction from hand written documents [17] and segmentation of dendritic spines from dendritic spine image [18], there is no pre-existing concept of flow pressure for object segmentation, especially in the context complementary pressure points created from two opposite flows (Please refer Fig 2). The complementary pressure points helps in deriving the optimum separation line between the overlapping objects. Also the fuzzyfication helped us to accurately estimate the pressure points. The pre-existing algorithms fail to segment overlapping objects of varying scale and resolution which is common scenario in case of microscopic cell images across different datasets.

Hypothetical water is allowed to flow over the planar image surface along a particular direction and it flows in that direction with some divergence angle. In the course of the flow, water wets those points on the image surface where flow could take place. As the objects act as obstacles protruding out of the image surface, water cannot flow over them. But water may flow bypassing the obstacles if there are paths beside them and may wet other side of the obstacle according to the divergence angle of the flow. In this case, water cannot wet (a) the image points on and within the obstacles and (b) the points just behind the obstacles, which do not come within the divergence angle of flow. Flow stops when it reaches the other end of the image. After the flow is over, the image looks like a wet surface on which there are multiple island-like structures because of the non-wetted object points and accompanying portion of the image surface where water cannot reach because of the obstructions caused by those objects. The island-like structures indicate of presence of objects, which are then easily segmented from the rest of the image surface. Due to the random orientation of nuclei in the image, water flows in eight different directions (east, north-east, north, north-west, west, south-west, west and south) from the image boundary points. A schematic representation of the method is presented in Fig 2. In the following we describe the fuzzy water flow in more formal way.

An image may be considered as a two dimensional (2D) grid of pixels, represented by {$Z^{2}|Z$ is the set of integers} [19]. A fuzzy object O is a fuzzy subset $\left\{\left(p,\mu_{O}\left(p\right)\right)|p\in Z^{2}\right\}$ of $Z^{2}$, where $\mu_{O}:Z^{2}\to [0,1]$ is the membership function. The support Θ(O) of an object O is the set of all pixels with non-zero membership value, i.e. $\Theta (O)=\{p\;|\;p\in Z^{2}\;\text{and}\;\mu_{O}\left(p\right)\neq 0\}$. Here, the input image is a binary image, consist of object pixels and background pixels. Let *B* denotes a set of pixels belongs to image border, *S* denotes the set of background pixels and *O* denotes the set of object pixels. A flow λ from *p* ∈ *B* is defined as a set of parameters {*p*, *α*, *β*, *ψ*(*p*)}, where *α* is the direction of the flow from *p*, *β* is the flow divergence angle and *ψ*(*p*) is the fuzzy flow-pressure function at *p*. *α* has eight different possible values 0°, 45°, 90°, 135°, 180°, 225°, 270°, and 315°. Let N*(*p*) denotes the set of eight neighbors of *p* excluding itself. Let *q*_1_, *q*_2_, *q*_3_, *q*_4_, *q*_5_, *q*_6_, *q*_7_, and *q*_8_ denotes the east, north-east, north, north-west, west, south-west, and south pixel to *p* respectively and *p*∠*q*_1_ = 0°, *p*∠*q*_2_ = 45°, *p*∠*q*_3_ = 90°, *p*∠*q*_4_ = 135°, *p*∠*q*_5_ = 180°, *p*∠*q*_6_ = 225°, *p*∠*q*_7_ = 270°, and *p*∠*q*_8_ = 315°. The fuzzy flow-pressure at a point *q* ∈ *S* and *q* ∈ N*(*p*) is defined as,

$$\begin{array}{c} \psi (q)=\{\begin{array}{cc} 1, & if\;q=p\\ \psi (p)cos(\theta -\alpha ), & otherwise. \end{array} \end{array}$$ (1)

**Fig 3:**
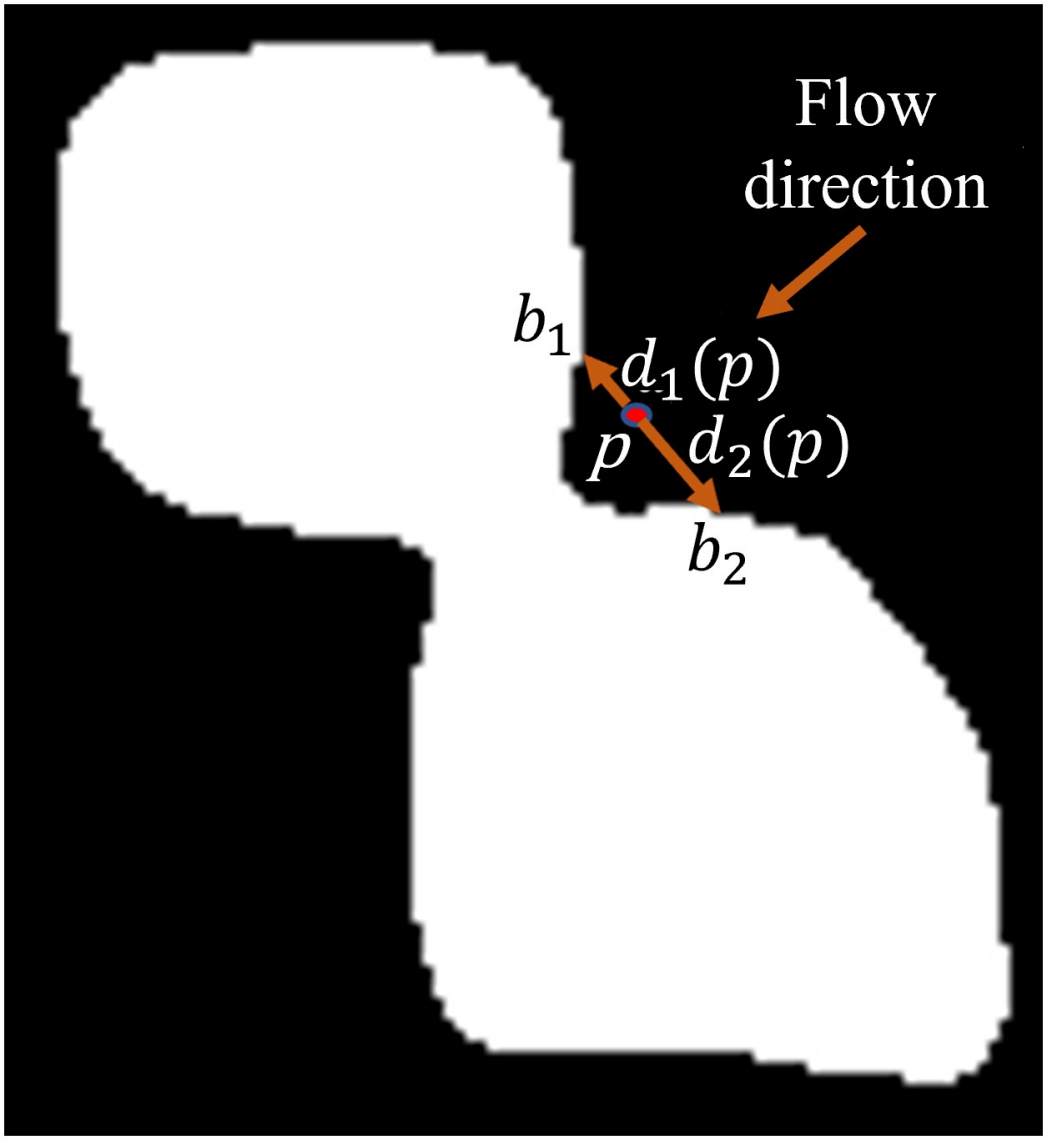
A constricted channel created by two overlapping nuclei. *p* is a point in the constricted channel. A line passing through *p*, orthogonal to the flow direction, connects two obstacle’s boundary point *b*_1_ and *b*_2_. *d*_1_(*p*) = distance of *p* from *b*_1_ and *d*_2_(*p*) = distance of *p* from *b*_2_.

Here *θ* is the direction of the vector joining *p* and *q*. *θ* can have 8 different values as explained above. Now, the flow λ is iteratively propagated from each wet point, *q* ∈ *W*, as {*q*, *α*, *β*, *ψ*(*q*)} and W is the set of wet pixels. We define obstacles as the set of object pixels, i.e. *O*, which may come in the line of the flow and create obstructions. Flow characteristics are changed based on the nature of the obstacles and the spatial distance of a point from the obstacle. In this context a channel may be defined as a region or passage in the line of the flow where the flow characteristics get changed because of the presence of nearby obstacles (see the pixelated image of Fig 2). For mathematical formulation of the variation of the fuzzy flow pressure within a channel, two distance metrics *d*_1_(*p*) and *d*_2_(*p*)) have been introduced for each *p* ∈ *W*. These two metrics are evaluated along the line through *p* in a direction orthogonal to the flow angle *α* to estimate the distance (in pixels) of the point *p* from the obstacle on either side of the flow point *p*. *d*_1_(*p*) and *d*_2_(*p*)) may or may not be equal as point *p* can be anywhere in the constricted channel. Please see Fig 3 for graphical explanation.

The constriction at point *q* is defined as *C*_*q*_ = *C*_*p*_ + Δ*C*, where, *C*_*p*_ is the constriction at the previous point with starting value as 0 (i.e. *C*_0_ = 0) and Δ*C* is the change in constriction with respect to the previous value. It is defined as $\Delta C=\frac{d_{max}\left(p\right)-d_{max}\left(q\right)}{max(d_{max}\left(p\right),d_{max}\left(q\right))}$, where, the *max*() function returns the maximum of the two arguments passed to it. The parameters *d*_*max*_(*p*) and *d*_*max*_(*q*) indicate the maximum distance of the flow point to the obstacle along the line orthogonal to the direction of the flow and are defined as *d*_*max*_(*p*) = *max*(*d*_1_(*p*), *d*_2_(*p*)) and *d*_*max*_(*q*) = *max*(*d*_1_(*q*), *d*_2_(*q*)). Δ*C* represents the fractional change in constriction compared to its previous flow point. Now, if *d*_*max*_(*p*) < *τ*; where *τ* is a heuristically chosen distance threshold, the fuzzy flow pressure at a neighboring flow point *q* may be redefined as

$$\begin{array}{c} \psi (q)=\{\begin{array}{cc} \emptyset (C_{q}), & \|p∠q\|=0\;\\ \emptyset (C_{q})cos(\theta -\alpha ), & otherwise. \end{array} \end{array}$$ (2)

where, $\emptyset (x)=\frac{1}{1+e^{-x}}$ represents a sigmoid function with unit slope parameter. With initial constriction, *C*_*p*_ = 0, the initial fuzzy flow pressure *ψ*(*p*) = ∅(*C*_*p*_) = ∅(0) = 0.5. As the sigmoid function ∅(*x*) returns value between 0 and 1, the value of *ψ*(*q*) lies between 0 and 1. *ψ* is a fuzzy subset of $R^{2}$.

The fuzzy flow pressure increases while entering into the channel. Now, based on the nature of the channel, it creates a pressure zone in that region. A pressure zone is digitally conceptualized as a cluster of neighboring points (*p*_*i*_) having *ψ*(*p*_*i*_) > 0.5, where ‘0.5’ is considered as the normal fuzzy flow pressure. The maximum pressure within a pressure zone is defined as pressure point and is identified as Ψ(*p*, *α*), where, *p*: *p*_*i*_ ∣ max(*ψ*(*p*_*i*_)) and *α* is the direction of flow. If two pressure points Ψ_1_(*p*_1_, *α*_1_) and Ψ_2_(*p*_2_, *α*_2_) are created such that ‖*α*_1_ − *α*_2_‖ = 180^0^, then the obstacle in between *p*_1_ and *p*_2_ is segmented if and only if ‖*p*_1_ − *p*_2_‖ ≤ *max*(*T*_*min*_N*(*p*_1_), *T*_*min*_N*(*p*_2_)), where *T*(*p*) is the measure of the thickness at a point *p*; $p\in \bar{\Theta }(O)$ and ‖⋅‖ is the Euclidean distance between two points. The fuzzy water flow routine runs for all the eight values *α* and the maximum value of *ψ* at any point *p* ∈ *S* is considered as the flow pressure at that point.

Let us define an object disconnectivity function at point (*x*, *y*) as *D*(*x*, *y*) = *T*(*x*, *y*)/2*τ* subject to maximum value of 1. Now, the normalized thickness at the pressure point is defined as the minimum *D*(*x*, *y*) of its neighboring obstruction points. If either of the two pressure points Ψ_1_(*p*_1_, *α*_1_) and Ψ_2_(*p*_2_, *α*_2_) has a value greater than the normalized thickness at any of the points *p*_1_ and *p*_2_, then a straight line is drawn between the pair of pressure points thereby segmenting the conjoint object.

## Results

In this section, we first describe the datasets used for experimental validation of our proposed segmentation pipeline. Next we evaluate the performance of our proposed method both qualitatively and quantitatively on the selected images from the datasets.

### Datasets description

Four public datasets have been used in this work namely NuCLS [20], MoNuSeg [21], TNBC [21], and S-BSST265 [22]. The NuCLS dataset contains over 220,000 labeled nuclei from breast cancer images from TCGA project. These nuclei were annotated through the collaborative effort of pathologists, pathology residents, and medical students. We used corrected single-rater dataset from whole the NuCLS dataset. This large annotated dataset were created by nonpathologists, and corrected by study coordinators under the supervision of a pathologist. It consists of 1744 images and 59,485 annotated nuclei. Images are of varying sizes between 287x292 and 830x765 pixels.

The second dataset MoNuSeg was published as a challenge dataset [21]. This multi-organ nuclei dataset consists of 30 training images and 12 separate test images. Each image dimension is 1024x1024 and around 22000 nuclei were manually annotated.

The third dataset, TNBC [21], consists of 50 Hematoxylin and eosin (H&E) stained images with 40x magnification from 11 different patients. Each image dimension is 512x512. Total 4022 cells are annotated in these images.

S-BSST265 dataset contains 79 annotated immunofluorescence (IF) images of different biological tissues and cells (ganglioneuroblastoma, neuroblastoma and others) of pathological and non-pathological origin used in imaging-based biomedical research. Multiple modalities (Zeiss Axioplan II, Zeiss- and Leica laser scanning microscopes (LSM)) were used for image acquisition while using different magnifications (10x, 20x, 40x, 63x objectives). The Nuclei in IF images were annotated and reviewed by trained pathologists and disease experts.

### Ablation study

**Fig 4:**
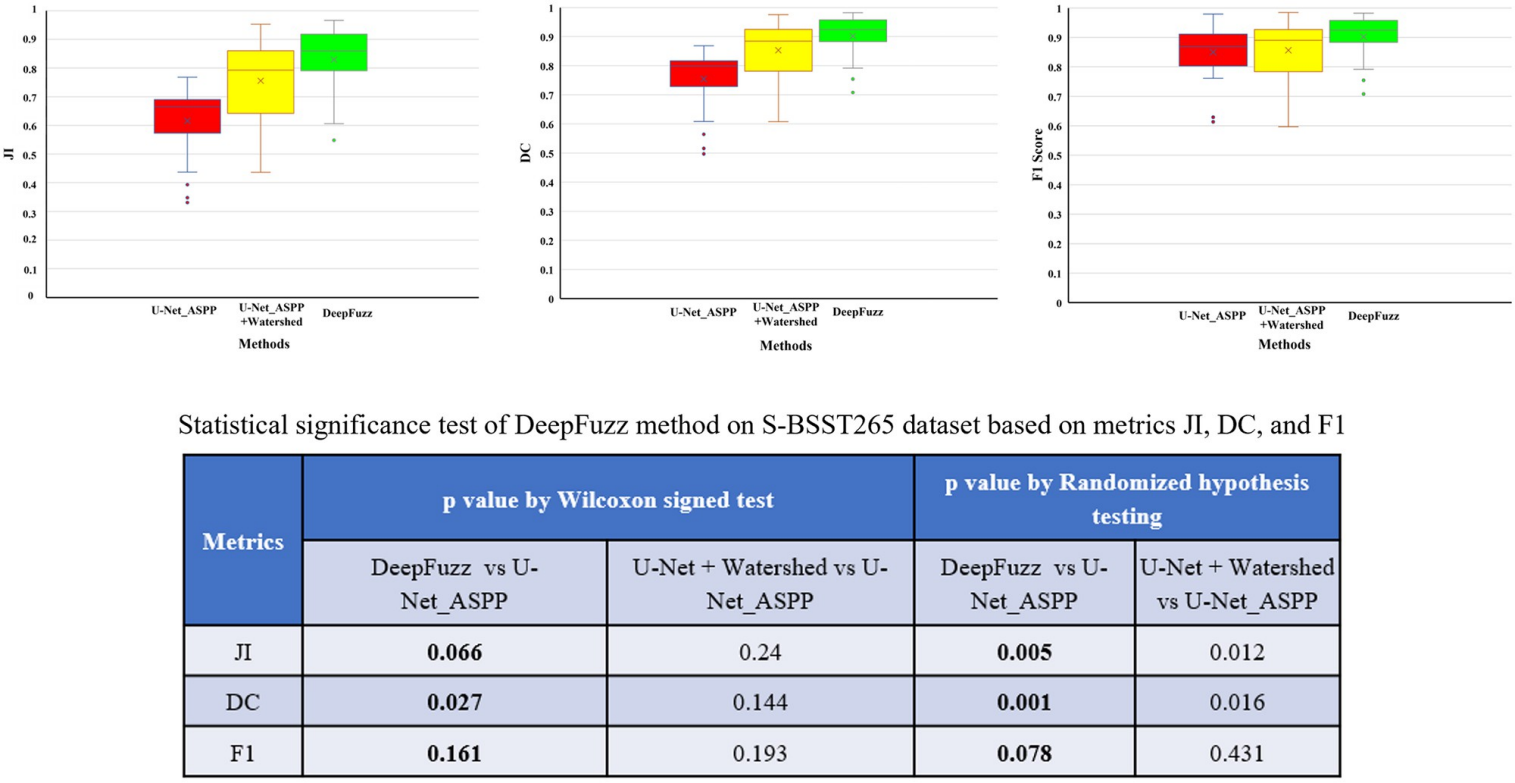
Box plots for comparative analysis of Deep-Fuzz with U-Net_ASPP and U-Net_ASPP + Watershed method on the test set of S-BSST265. The left panel shows Jaccard Index (JI), the middle panel shows Dice Coefficient, and the right panel shows F1 score. The dots outside the box denote outliers. The table shows the *p* values considering U-Net_ASPP as the base model. The test was done using Wilcoxon signed test and Randomized hypothesis testing.

In this sub section, we performed several experiments to validate the reason of choosing different modules in the proposed segmentation method Deep-Fuzz. After achieving the coarse nuclei segmentation from base framework we further apply our novel fuzzy water flow method to segment the overlapping nuclei. We compared the segmentation performance of our proposed fuzzy water flow method with the marker controlled watershed method [23] in segmenting overlapped nuclei. We used binary distance transform to automatically generate the seed points/marker to run the watershed algorithm. For the ablation study we have used S-BSST265 data set. We examined how the segmentation performance varies in terms of few standard metrics used to evaluate segmentation with exclusion and inclusion of different modules with the base deep learning network. Table 1 describes the evaluation metrics used in short. It is evident from Table 2 that the proposed fuzzy water flow method with the base framework outperforms the base framework with traditional watershed segmentation method. We also tested the statistical significance of the improvement in result by Deep-Fuzz using the method Wilcoxon signed test and Randomized Hypothesis testing. We showed statistical significance in terms of Dice coefficient (DC), Jaccard index (JI), and F1 score which are the main indicators of the performance of a segmentation model. Wilcoxon signed test is not always valid for complex metrics like DC, JI, and F1 [24]. We have also visually represented the comparative performance of Deep-Fuzz using Box-Whisker Plot. Please see the Fig 4.

**Table 1:** Description of the evaluation metrics: Jaccard Index (JI), Dice Coefficient (DC), Volume Similarity (VS), Accuracy (Acc), Precision (Pre), Recall (Rec), F1, and F2. TP: True Positive, FP: False Positive, TN: True Negative, FN: False Negative.

Metrics	Description	Metrics	Description
**JI**	$\frac{TP}{TP+FP+FN}$	**Pre**	$\frac{TP}{TP+FP}$
**DC**	$\frac{2TP}{2TP+FP+FN}$	**Rec**	$\frac{TP}{TP+FN}$
**VS**	$1-\frac{\left|FN-FP\right|}{2TP+FP+FN}$	**F1**	$\frac{2\times Recall\times Precision}{Precision+Recall}$
**Acc**	$\frac{TP+TN}{TP+TN+FP+FN}$	**F2**	$\frac{2\times Recall\times Precision}{4\times Precision+Recall}$

**Table 2:** Segmentation performance of the backbone network with different modules. The backbone network is U-net.

U-Net	ASPP	Watershed technique	Fuzzy Flow	JI	Dice	VS	Acc	Pre	Rec	F1	F2
✓	✓	-	-	0.616	0.754	0.772	0.624	0.776	0.906	0.850	0.893
✓	✓	✓	-	0.755	0.853	0.922	0.871	**0.911**	0.822	0.856	0.834
✓	✓	-	✓	**0.833**	**0.904**	**0.964**	**0.906**	0.906	**0.907**	**0.904**	**0.906**

### Quantitative results

**Fig 5:**
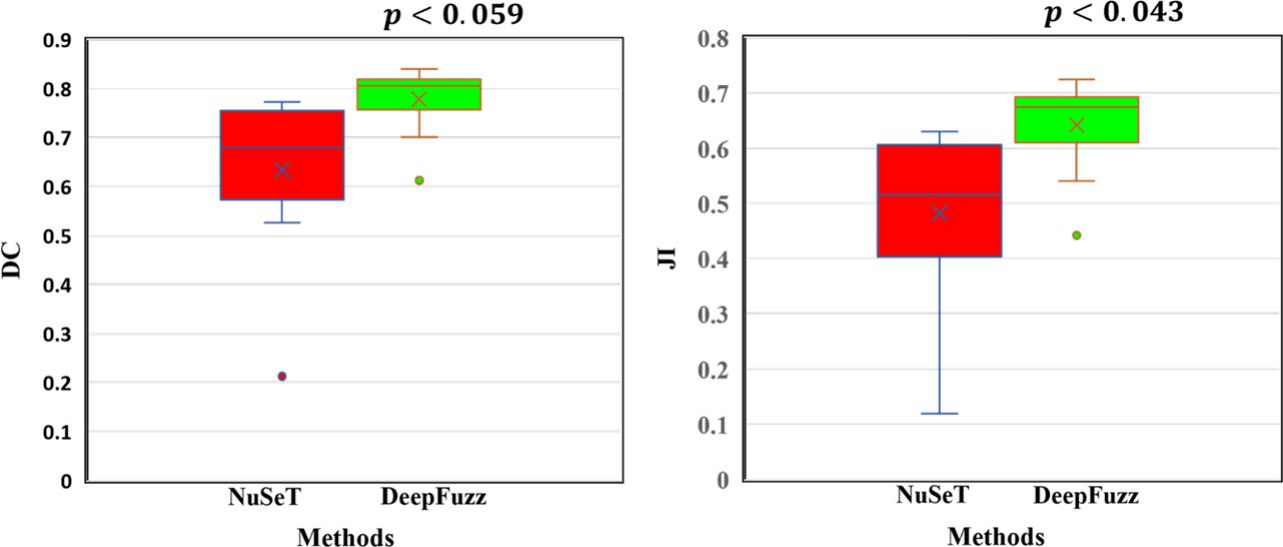
Box Whisker plots for comparative analysis of Deep-Fuzz with NuSeT [28] method on the test set of TNBC. The left panel shows the Dice coefficient and the right panel shows Jaccard index. The p values for DC and JI were calculated using randomized hypothesis testing. The p-values denote the statistical significance of the Deep-Fuzz method over the NuSeT method in terms of DC and JI.

We have used multiple evaluation metrics to show the goodness of the proposed Deep-Fuzz method. One metric is not enough to evaluate the performance of the segmentation models. For example, Accuracy can be misleading when there are only a few target object pixels (nuclei) and mostly background pixels in the image. The measure will be biased in mainly reporting how well the model identifies background. Precision measures among all the pixels predicted as belonging to the target object(nuclei), how many actually belong to the target object (nuclei) while Recall captures among all the pixels annotated as object pixels (nuclei) how many pixels the model predicts as object pixels (nuclei). Precision gives the accuracy of the positive prediction while recall tells us about the completeness of the positive result. There exists a trade-off between Precision and Recall. Now among precision and recall which one to be prioritized that depends on the specific use case. Jaccard Index and Dice Coefficient are similarity measures widely used in the performance evaluation of the segmentation models. Jaccard Index and Dice Coefficient both measure degree of overlap between ground truth and predicted image and they are positively correlated. Volume similarity does not consider the overlap between ground truth and the predicted image. It compares the similarity of total volume in the predicted image and the ground truth image. F1 score represents the harmonic mean of Precision and Recall and give equal weightage to the both. F2 gives more weightage to the Recall than Precision. So F2 is used instead of F1 where Recall is more important than precision. In all the image datasets, we initially randomly selected 10% images for testing purpose (except MoNuSeg) and later to train the model we randomly separated remaining images into training(80%) and validation(20%). In Nucls dataset, among 1744 images, 174 images and their corresponding ground truth were randomly separated for testing. From the remaining images i.e., 1395(80%) images were randomly selected to train the base model and 175 were used (10%) images for validation purpose. All the images were resized to 512x512. The MoNuSeg, training data set contains 30 images with 22,000 nuclear boundary annotations. 24 (10%)images were randomly selected to train the network and 6 images for the validation. 14 test images were given separately for this dataset. All the images of MoNuSeg dataset were resized to 512x512. The TNBC(Triple Negative Breast Cancer) dataset contain 50 H&E stained images with 512x512 resolution. All images in this dataset were extracted from 11 TNBC patients with multiple cell types including endothelial cells, inflammatory cells, and myoepithelial breast cells. We used 11 images from randomly selected 3 different patients for testing purpose. From the remaining 8 patients, 32 images were selected for training and 7 images for validation. In S-BSST265 data set among 79 IF images in the same way we selected 64 images for training purpose, 8 images for validation and 7 images for testing purpose. The images were resized to 512x512. For training the framework, we used ‘Adam’ optimizer with learning rate of 0.007 and Dice loss as the loss function. The framework was trained for 100 epochs for each dataset. Segmentation task is challenging in nuclei images because of their large variances in appearance as compared to other organs. The most difficult part is segmentation of overlapping nuclei. The issue of overlapping nuclei segmentation is not addressed well in the literature. Although the base network with tuned hyper-parameters gives satisfactory segmentation result on all of the four data sets, the problem of overlapping nuclei segmentation is not achieved well. We calculated percentage of overlapping nuclei from all of the data sets after phase-I segmentation. We found 22%, 15%, 19% and 18% overlapping nuclei in the NuCLS, MoNuSeg, TNBC, and S-BSST265 data set respectively. The proposed fuzzy water flow algorithm is applied on the segmented images from the phase-I to further segment the overlapping nuclei. The comparison of the quantitative performance of our proposed segmentation pipeline is shown in Table 3. To the best of our knowledge, no other work has been reported on NuCLS data set before. In MoNuSeg dataset, we compared the results of Deep-Fuzz with three other state of the art methods [25–27]. Although Deep-Fuzz underperformed in comparison with the other methods in terms Acc, Pre, Rec, F1 and F2, it performed slightly better in terms of JI and DC which are considered to be the main indicators of segmentation performance. In TNBC dataset, Deep-Fuzz results are compared with two other methods [28, 29]. In [28], they have used a hybrid network comprises of U-Net and Region Proposal Network as deep learning module and marker controlled watershed as postprocessing step to separate overlapping nuclei. We implemented the NuSeT code available in the GitHub repository given in the paper. It is evident form Table 3 that Deep-Fuzz outperforms NuSeT in terms of all the evaluation metrics. We also evaluated the statistical significance of the improvement in results by Deep-Fuzz with respect to NuSeT in terms of JI and DC. We used randomized hypothesis testing for evaluating statistical significance and achieved *p* value of 0.043 for JI and 0.059 for DC. Fig 5 visually compares the Deep-Fuzz and NuSeT method in terms of JI and DC using Box Whisker Plot. Kong et. al. [29] used a two staged stacked U-Net with attention mechanism for nuclear segmentation in histopathology images. They reported only JI and F1. In addition to the reported evaluation parameters, we also calculated the Power of the Deep-Fuzz method i.e., the probability of detecting True Positives (TP). The Power or Pr(TP) is defined as 1 − *β*, where *β* is the probability of detecting Type II errors i.e., False Negatives (FN). In the case of NuCLS, MoNuSeg, TNBC, and S-BSST265 datasets the calculated values are 0.28, 0.29, 0.17, and 0.09 respectively. So the Power values in the case of NuCLS, MoNuSeg, TNBC, and S-BSST265 datasets are 0.72, 0.71, 0.83, and 0.91 respectively.

**Table 3:** Comparative analysis of the segmentation performance of the proposed Deep-Fuzz in terms of JI, DC, VS, Acc, Pre, Rec, F1 and F2 on NuCLS, MoNuSeg, TNBC, and S-BSST265 data set respectively. **Bold text**: Best Result.

Dataset	Models	JI ↑	DC ↑	VS ↑	Acc ↑	Pre ↑	Rec ↑	F1 ↑	F2 ↑
**NuCLS**	Deep-Fuzz	0.631	0.772	0.912	0.716	0.844	0.716	0.693	0.661
**MoNuSeg**	Deep-Fuzz	**0.661**	**0.791**	0.931	0.759	0.790	0.714	0.745	0.725
	GCT [25]	0.640	0.777	-	0.907	0.922	**0.854**	**0.887**	**0.867**
	CAC [26]	0.645	0.782	-	0.910	0.930	0.828	0.876	0.844
	[27]	0.656	0.789	-	**0.914**	**0.933**	0.842	0.885	0.859
**TNBC**	Deep-Fuzz	**0.642**	**0.778**	**0.907**	**0.756**	**0.756**	**0.825**	0.778	**0.802**
	NuSeT [28]	0.482	0.634	0.824	0.662	0.661	0.694	0.634	0.642
	[29]	0.621	-	-	-	-	-	**0.806**	-
**S-BSST265**	Deep-Fuzz	0.833	0.904	0.964	0.906	0.906	0.907	0.904	0.906
	[22]	-	**0.912**	-	-	-	-	-	-

### Qualitative result

**Fig 6:**
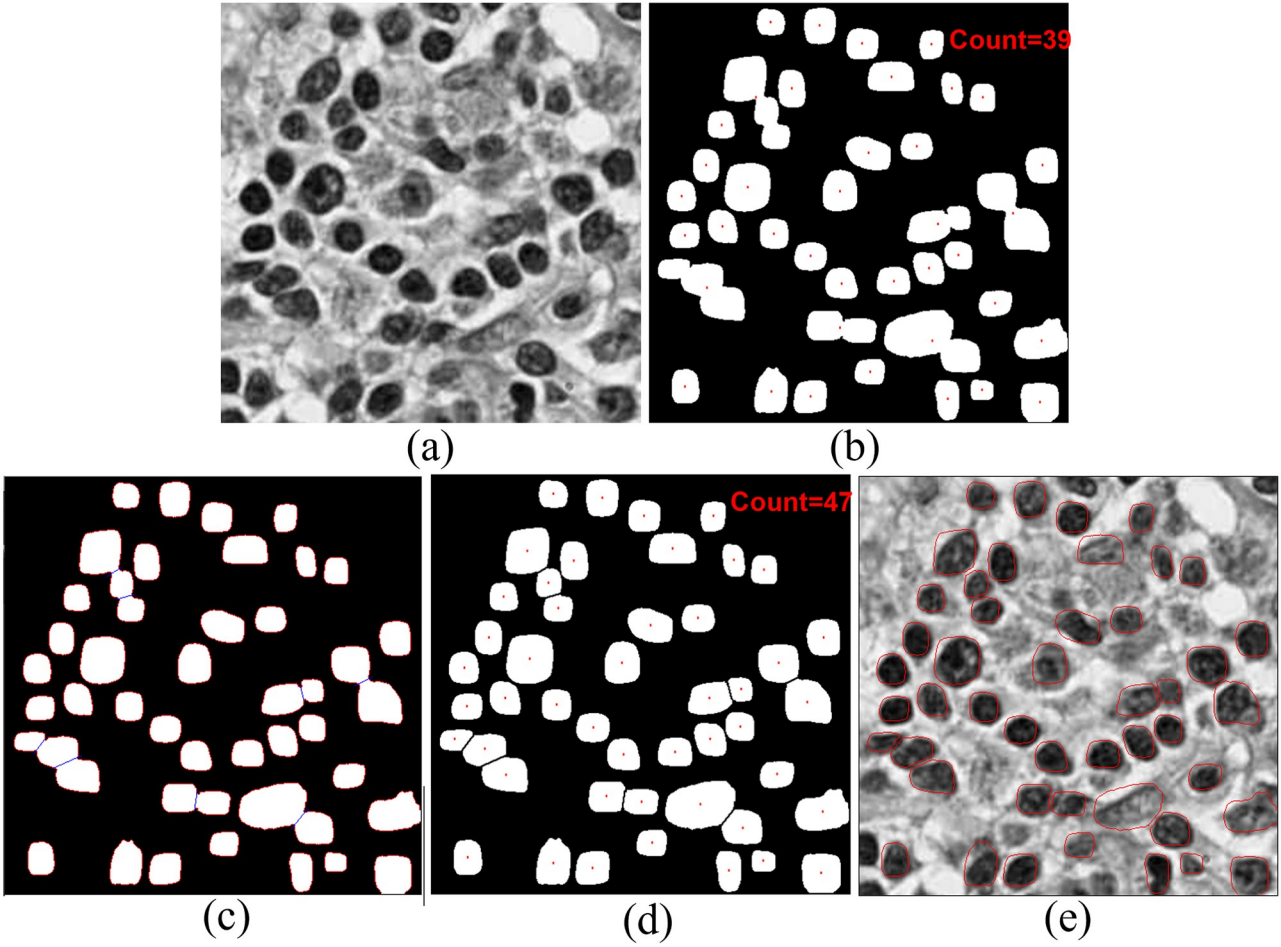
(a) Original Image (b) Segmented nuclei image with base framework. Red dots are CG points of the segmented nuclei. Total nuclei count = 39 (c) Separation line (blue) generated by the proposed fuzzy water flow algorithm between overlapping nuclei (d) Final segmentation. Total nuclei count = 47 (e) Overlaying of final segmentation boundary with original image.

**Fig 7:**
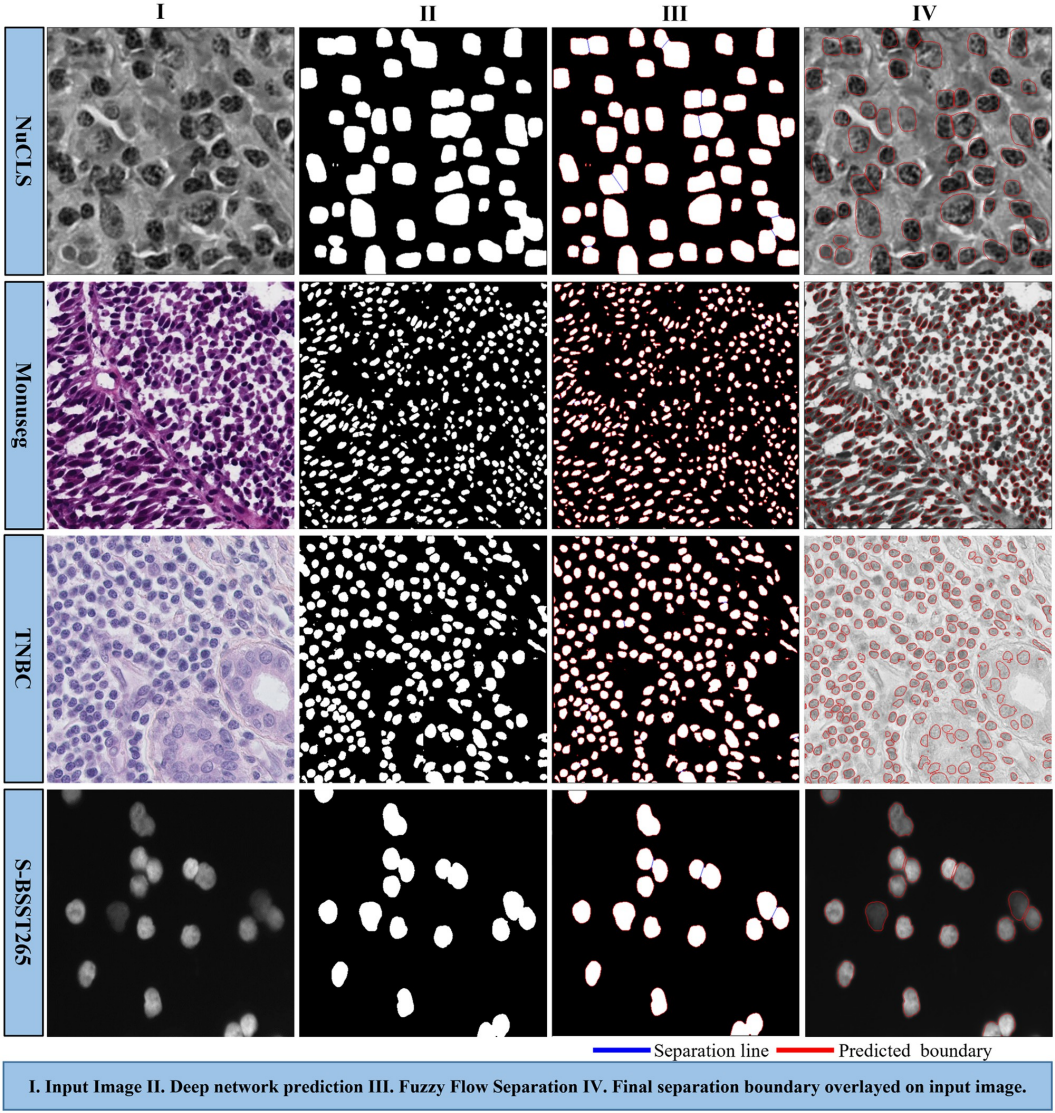
Deep-Fuzz segmentation results on sample data from four public datesets: NuCLS, Monuseg, TNBC and S-BSST265.

**Fig 8:**
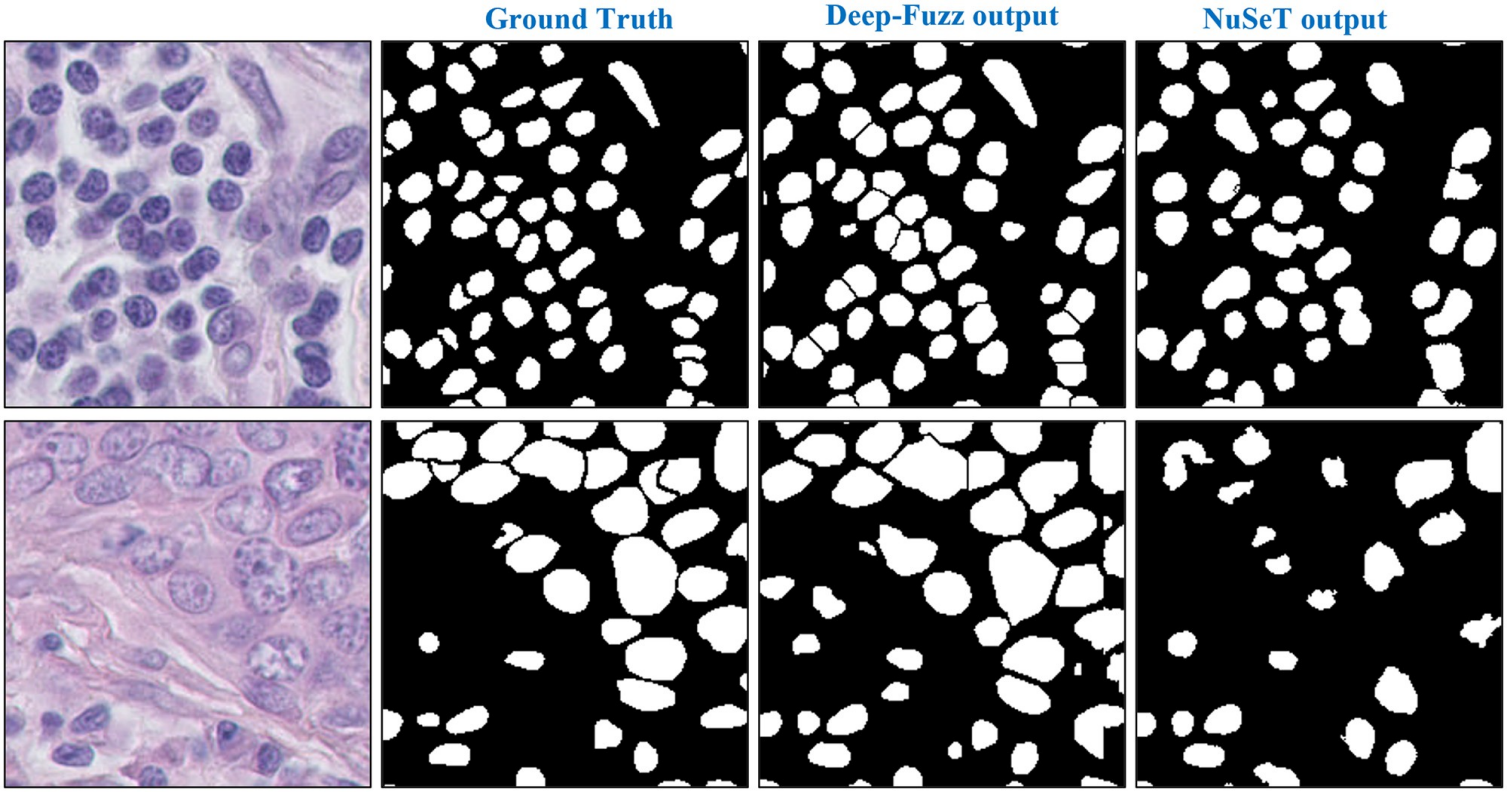
Qualitative comparison of the Deep-Fuzz output with the state-of-the-art NuSeT [28] method on sample images from TNBC dataset.

**Fig 9:**
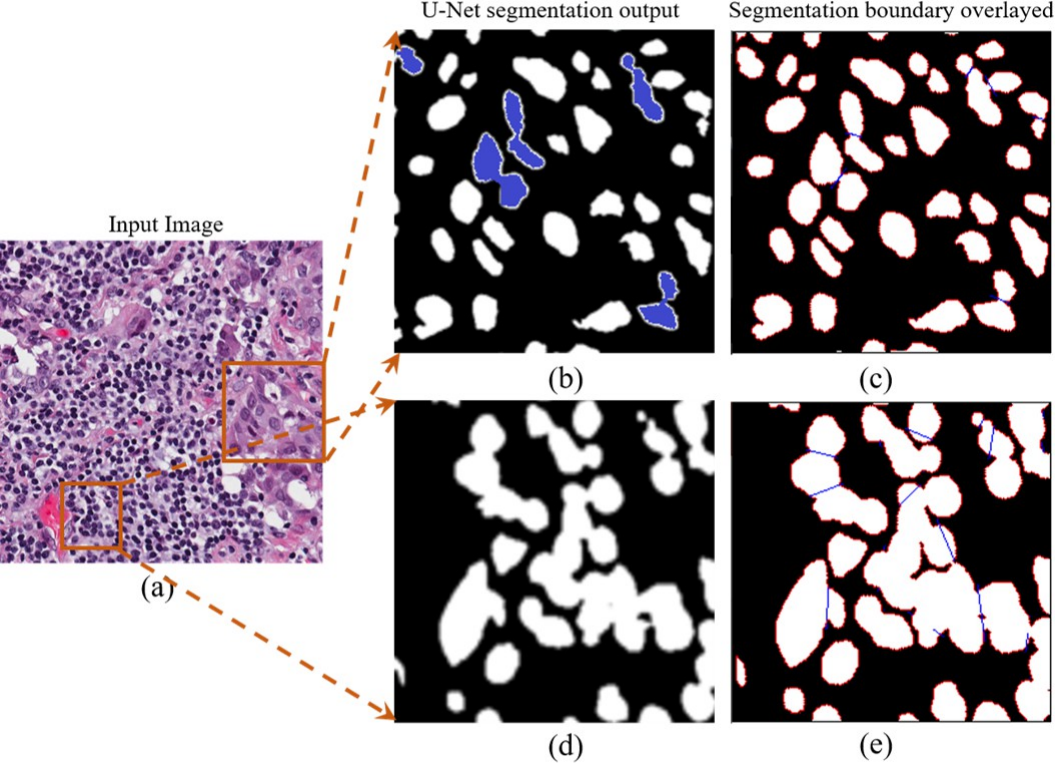
An example of successful (top row, b-c) and unsuccessful (bottom row, d-e) segmentation in an image sample from the MoNuSeg dataset. (a) Input image with two regions of interest (ROI) (b) Magnified segmentation output of the selected ROI by U-Net_ASPP model. Overlapping cells are marked with blue color. (c) A thin blue separation line is created between overlapping nuclei by our proposed fuzzy water flow algorithm. (d) Magnified segmentation output of the selected ROI by U-Net_ASPP model. Overlapping nuclei formed a clump which shows the failure of U-Net_ASPP. (e) Our proposed fuzzy water flow algorithm successfully separates some of the overlapping nuclei, but fails separate under-segmented nuclei, forming a clump. Few incorrect separation lines are generated.

In Fig 6(b) we qualitatively represent how the proposed Deep-Fuzz method provides more accurate count of the total number of nuclei present in an image. In the selected sample from the NuCLS dataset the ground truth image contains 47 nuclei annotations. After segmentation using the base framework, we can see many segmented nuclei are overlapped with each other (see Fig 6(c)) and total number of segmented nuclei in this image are now 39. After applying fuzzy water flow algorithm, we get a blue separation line between the overlapped nuclei (see Fig 6(d).) Fig 6(e) shows the final segmentation result and the total number of nuclei in the final segmented images is 47. Fig 7 depicts qualitative segmentation results on Nucls, Monuseg, TNBC and S-BSST265 datasets respectively. The final segmentation boundaries are overlayed on the original image. Fig 8 presents qualitative comparison of Deep-Fuzz results with the results achieved by NuSeT [28] on two sample images from TNBC dataset. We can see NuSeT failed to segment many nuclei in both of the samples and also produced incorrect segmentation in many places. Deep-Fuzz produces better segmentation result. It is observable from Fig 8 that Deep-Fuzz also outperforms NuSeT in overlapping nuclei segmentation. In Fig 9, we also show illustrative examples where the flow algorithm succeeds and fails at segmenting challenging regions of interest using a sample image from the MoNuSeg dataset. The proposed fuzzy water flow algorithm successfully segments overlapping nuclei when they are morphologically separable (see Fig 8(a)). If the overlapping nuclei are morphologically separable then only the constricted channels will be created by the nuclei boundaries and eventually maximum flow pressure points will be generated. It fails to separate overlapping nuclei or draw inaccurate separation lines where they are morphologically inseparable and are forming a clump (see Fig 8(e)).

## Discussion

The proposed Deep-Fuzz method provides satisfactory cell nuclei segmentation result on four publicly available datasets. From the literature we can see that the U-Net is a popular deep network used in medical image segmentation task. Here, we used ASPP block in the encoder-decoder bottleneck instead of using traditional max pooling method. ASPP block extracts multi-scale information by varying the size of receptive field. From the ablation study we can see that the U-Net with ASPP block gives the best result. In the next phase we compared the result our proposed fuzzy water flow algorithm with traditional watershed algorithm in segmentation of overlapping nuclei.

Base deep network in combination with fuzzy water flow gives better overall segmentation performance than base deep network followed by traditional watershed algorithm. The power of the Deep-Fuzz method comes from the synergy between deep learning and proposed fuzzy water flow. Deep learning overcomes the limitation traditional feature engineered methods and help in better segmentation of the nuclei from the background but it fails to separate overlapped nuclei from each other. From qualitative and quantitative result presented it evident that our proposed fuzzy water flow method successfully segments overlapped nuclei in any arbitrary orientation and scale. Fuzzy water flow method fails to segment overlapped nuclei at few places where they are morphologically inseparable and forming a clump. In future, the proposed fuzzy water flow method may be further improved to better estimate the separation channel between overlapped nuclei and to compute more accurate separation line.

In Table 3, we can see that in the case of MoNuSeg dataset, Deep-Fuzz fails to beat the state of the art methods in terms of all other evaluation parameters except JI and DC. The methodological contribution of Deep-Fuzz is in developing the fuzzy waterflow algorithm, that works in conjunction with the deep segmentation algorithm, applied in phase-1. In our work, we have used a fast and light-weight U-Net “Deep” module to generate the coarse segmentation result, which is fed to the phase-2 “Fuzz” module to further segment the conjoint cell nuclei, that were otherwise non-separable using the conventional “Deep” module. We present this as a validation that when a conventional “Deep” module fails to segment conjoined structures, our novel “Fuzz” module may come to the rescue. We are aware of the performance limitations of the phase-1 “Deep” module, and future research is focused on improvement of the same. However, we have shown in our results that even with the fast and light-weight “Deep” module, the final segmentation results are comparable with the state-of-the-art. More specifically, our “Fuzz” module starts from a point when the conventional “Deep” modules fail. Therefore, we further argue that our proposed method may not be compared only in terms of performance metrics, but also in terms of applicability in segmentation of otherwise difficult cases. This also validates the general need of hybrid strategies for the solution of complex segmentation problems in medical imaging. The “Fuzz” module has some clear advantages in comparison with the other methods used in phase-2 generally. For overlapping nuclei segmentation in the phase-2, most of the works used marker controlled watershed or level set methods [21, 28] for generation of separation boundary which requires initial seed points for initiation. The final accuracy of those methods depends on the accuracy of the seed selection method. The fuzzy water flow algorithm does not require any seed initialization.

## Conclusion

The proposed Deep-Fuzz method advocates the importance of synergistic integration of the data driven deep learning and hand-formulated image processing simultaneously, and presents a novel practical implementation to achieve that. The proposed fuzzy water flow scheme is a fast and efficient unsupervised method that improves upon the coarse output of a lightweight U-Net backbone. The resulting pipeline produces state-of-the-art results on four benchmark cell nuclei segmentation datasets and outperforms several recent competing methods for TNBC dataset. From the perspective of interpretable machine learning, the transparent design of the fuzzy water flow scheme grounds the method mathematically, and thus for the discerning reader, the authors hope that it furthers the cause of trustworthy machine learning.
