# Correction: Deep-Fuzz: A synergistic integration of deep learning and fuzzy water flows for fine-grained nuclei segmentation in digital pathology

**DOI:** 10.1371/journal.pone.0295111

**Published:** 2023-11-27

**Authors:** Nirmal Das, Satadal Saha, Mita Nasipuri, Subhadip Basu, Tapabrata Chakraborti

The Data Availability link for this paper is incorrect. The correct link is: https://github.com/dasnirmal/DeepFuzz

There is an error in affiliation 3 for author Satadal Saha. The correct affiliation 3 is: Department of Electronics and Communication Engineering, MCKV Institute of Engineering, Howrah, West Bengal, India

## References

[1] Das N, Saha S, Nasipuri M, Basu S, Chakraborti T. (2023) Deep-Fuzz: A synergistic integration of deep learning and fuzzy water flows for fine-grained nuclei segmentation in digital pathology. PloS ONE, 18(6): e0286862. 10.1371/journal.pone.0286862 37352172 PMC10289330

